# Involvement of Iliofemoral Arteries in PET/CT Is Associated with Atherosclerotic Risk Factors in Takayasu’s Arteritis

**DOI:** 10.3390/jcm14238607

**Published:** 2025-12-04

**Authors:** Sema Kaymaz-Tahra, Salih Ozguven, Aysegul Avcu, Nuh Filizoglu, Ali Ugur Unal, Tunc Ones, Tanju Yusuf Erdil, Fatma Alibaz-Oner, Haner Direskeneli

**Affiliations:** 1 Division of Rheumatology, Department of Internal Medicine, School of Medicine, Bahcesehir University, 34732 Istanbul, Turkey; 2 Department of Nuclear Medicine, School of Medicine, Marmara University, 34854 Istanbul, Turkey; drsozg@gmail.com (S.O.); nuhfilizoglu@gmail.com (N.F.); tones@marmara.edu.tr (T.O.); yerdil@marmara.edu.tr (T.Y.E.); 3 Division of Rheumatology, Department of Internal Medicine, School of Medicine, Marmara University, 34854 Istanbul, Turkey; aysegulavci.89.99@gmail.com (A.A.); aliugurunal@hotmail.com (A.U.U.); falibaz@gmail.com (F.A.-O.); hanerdireskeneli@gmail.com (H.D.)

**Keywords:** autoimmune diseases, vasculitis, Takayasu arteritis, cardiovascular risk factors

## Abstract

**Background/Objectives**: Takayasu’s arteritis (TAK) is an inflammatory vascular disease, but atherosclerotic mechanisms may also contribute to vascular injury in TAK. This study aimed to evaluate the clinical and imaging characteristics of TAK patients with and without iliofemoral artery involvement on FDG-PET/CT, focusing on the association with classical atherosclerotic risk factors. **Methods**: Patients fulfilling the 1990 ACR classification criteria for TAK who underwent FDG-PET/CT imaging during follow-up were retrospectively analyzed. Demographic, clinical, and laboratory data were recorded, including traditional cardiovascular risk factors: diabetes, hypertension, hyperlipidemia, smoking, obesity (BMI ≥ 30 kg/m^2^), and family history of cardiovascular disease. PET vascular activity score (PETVAS) and visual analysis were used to assess vascular inflammation. **Results**: PET/CT scans of 77 TAK patients (F/M = 63/14) were evaluated. The mean age was 43.0 ± 12.9 years, and the mean disease duration was 120.1 ± 88.8 months. Iliofemoral artery involvement was observed in nine (12%) patients. Compared to those without such involvement, these patients were older (52.5 ± 17.4 vs. 41.3 ± 12.1 years, *p* = 0.098), more frequently male (44% vs. 6%, *p* = 0.015), and had higher CRP levels (38.5 mg/L vs. 10.7 mg/L, *p* = 0.026). Smoking (77% vs. 40%, *p* = 0.045) and chronic kidney disease (22% vs. 4%, *p* = 0.046) were also more prevalent. PET activity according to visual analysis was higher among those with iliofemoral involvement (67% vs. 27%, *p* = 0.015). In multivariate analysis, older age (OR = 1.07, *p* = 0.044) and male sex (OR = 6.68, *p* = 0.039) were independently associated with iliofemoral artery involvement. **Conclusions**: Iliofemoral artery involvement on PET/CT in TAK patients was associated with traditional atherosclerotic risk factors—particularly older age, male sex and smoking. These findings suggest that atherosclerotic mechanisms may coexist with or amplify vascular inflammation in TAK. Aggressive management of cardiovascular risk factors should therefore be emphasized in this subgroup of TAK patients.

## 1. Introduction

Takayasu’s arteritis (TAK) is a chronic, large-vessel vasculitis that predominantly affects the aorta and its major branches, leading to stenotic or aneurysmal vascular lesions [1]. Although inflammation is the key pathogenic mechanism in TAK, accumulating evidence indicates that atherosclerosis and vasculitis may overlap, especially in patients with traditional cardiovascular risk factors. It was reported that atherosclerotic plaques were significantly more frequent among patients with TAK compared with healthy controls, suggesting an interaction between chronic inflammation and accelerated atherosclerosis [2,3].

Atherosclerotic changes tend to become more common as patients get older and the disease lasts longer, making imaging findings harder to interpret. Prolonged disease duration has been associated with atherosclerosis in TAK [4], and in older patients, atherosclerotic vascular uptake, often in the abdominal aorta and iliac/femoral beds, can overestimate inflammatory activity on 18F-fluorodeoxyglucose positron emission tomography/computed tomography (FDG-PET/CT) [5,6].

FDG-PET/CT is a valuable modality for assessing vascular inflammation in TAK, aiding both diagnosis and monitoring. However, its specificity is limited, as FDG uptake may also occur in metabolically active atherosclerotic plaques [5]. This diagnostic overlap is especially relevant in patients with cardiovascular comorbidities where vascular uptake may not necessarily indicate active vasculitis.

Furthermore, iliofemoral artery involvement is well-recognized as a hallmark of advanced atherosclerotic disease in the general population [7]. Therefore, when detected in TAK, such involvement requires careful interpretation and further study to identify whether it represents inflammation, atherosclerotic remodelling, or both.

In the present study, we aimed to compare the demographic, clinical, and imaging characteristics of TAK patients with and without iliofemoral artery involvement on PET/CT, with a particular focus on the association between this finding and traditional atherosclerotic risk factors.

## 2. Materials and Methods

Patients (*n* = 77) who were diagnosed with TAK according to American College of Rheumatology (ACR) 1990 classification criteria [8] and underwent FDG PET/CT imaging during their routine follow-up were evaluated, in this non-interventional, observational study. Clinical and imaging data was recorded from patient charts. PET/CT data is collected between January 2015 and January 2025. The study was approved by Marmara University local Ethical Committee (No: 09.2019.605). All patients provided written informed consent.

Physician’s Global Assessment (PGA) was used to determine clinical activity according to Kerr (NIH) criteria. Clinical activity was assessed using the NIH/Kerr criteria, incorporating systemic and vascular symptoms, ESR, CRP, and imaging findings [1]. Laboratory parameters (ESR, CRP) were collected within ±7 days of PET/CT scanning.

Immunosuppressive (IS) and glucocorticoid (GC) uses and the doses of the treatments were recorded. GC doses were given in prednisolone equivalent doses. Because GC therapy and immunosuppressive exposure may suppress FDG uptake independent of disease activity both GC dose (prednisone-equivalent) and IS use at the time of PET/CT were included as covariates in sensitivity analyses. PET-based vascular activity (VA) was evaluated separately from PGA because the two metrics represent distinct biological constructs.

The association between iliofemoral involvement and traditional atherosclerotic risk factors including age, sex, hypertension, diabetes, hyperlipidemia, smoking were evaluated. Smoking was defined as current or ever smoking; dyslipidemia as LDL ≥ 130 mg/dL or use of lipid-lowering therapy; and obesity as BMI ≥ 30 kg/m^2^. The definition of chronic kidney disease (CKD) was made as eGFR < 60 mL/min/1.73 m^2^.

### 2.1. FDG-PET/CT Imaging Technique

All patients underwent PET/CT imaging after fasting for a minimum of six hours, during which only non-glucose oral hydration was permitted. The scans were performed following the joint procedural recommendations of the European League Against Rheumatism (EULAR), the European Association of Nuclear Medicine (EANM), the Society of Nuclear Medicine and Molecular Imaging (SNMMI), and the PET Interest Group (PIG), and were endorsed by the American Society of Nuclear Cardiology (ASNC) [9,10].

Before FDG administration, patients’ blood glucose levels were confirmed to be below 126 mg/dL. After receiving 3.7 MBq (0.1 mCi)/kg of FDG intravenously, patients rested for approximately one hour in a quiet, temperature-controlled environment (20–22 °C). According to institutional protocol, oral but not intravenous contrast was used.

Imaging was performed on an integrated PET/CT scanner (Discovery-16LS, GE Healthcare, Chicago, IL, USA). PET data were acquired in 3D mode from the mid-skull to below the knees and reconstructed in transverse, coronal, and sagittal planes. A low-dose CT scan was obtained for attenuation correction and anatomical localization. The resulting images were then transferred to a dedicated workstation (Advantage Windows 4.5, GE Healthcare) for further analysis and interpretation.

### 2.2. Image Interpretation

Two independent nuclear medicine physicians, blinded to all clinical data, interpreted the PET/CT images. A total of 15 arterial territories were evaluated, including the ascending aorta, aortic arch, descending thoracic aorta, abdominal aorta, innominate, carotid, subclavian, axillary, iliac, and femoral arteries. For PETVAS scoring, which was performed one hour after FDG administration, nine arterial regions (ascending aorta, aortic arch, descending thoracic aorta, abdominal aorta, right and left carotid arteries, innominate artery, and right and left subclavian arteries) were rated on a 0–3 scale according to FDG uptake intensity, yielding a total score (range: 0–27). FDG uptake was graded as follows: grade 0 = no uptake; grade 1 = uptake lower than liver; grade 2 = uptake equal to liver; and grade 3 = uptake higher than liver [11].

Visual analysis (VA) using hepatic FDG uptake as a reference was also performed. Arterial regions showing uptake equal to or exceeding that of the liver (≥grade 2) were considered ‘active’ or ‘positive.’ An ‘active PET/CT’ was defined as the presence of at least one active arterial segment.

To differentiate active vasculitic lesions from atherosclerotic uptake, arterial FDG uptake was assessed according to the pattern, intensity, and distribution of the signal. Uptake was considered suggestive of vasculitic activity when it was circumferential or segmental along the arterial wall and equal to or higher than the liver activity, in accordance with the joint procedural recommendations of EANM/EULAR/SNMMI [10]. In contrast, focal or eccentric uptake confined to arterial bifurcations or calcified segments was regarded as atherosclerotic and was not classified as active vasculitis. During both PETVAS calculation and visual activity assessment, vascular territories showing uptake patterns compatible with atherosclerosis were excluded and not scored as active disease

### 2.3. Statistical Analysis

All statistical analyses were conducted using SPSS version 22.0 (IBM Corp., Chicago, IL, USA). Continuous variables were expressed as mean ± standard deviation or median (minimum–maximum), according to data distribution. Categorical variables were compared using the chi-square or Fisher’s exact test. Independent continuous variables were compared with the Mann–Whitney U test. A *p*-value < 0.05 was considered statistically significant. Logistic regression analysis was applied to identify variables independently associated with iliofemoral artery involvement. Variables with a *p*-value < 0.05 in the univariate analysis were entered into the multivariate analysis.

Univariate analyses were performed separately according to the analytical level of each variable. Scan-level predictors (e.g., PET-derived parameters) were analyzed using simple binary logistic regression, whereas patient-level variables (e.g., demographic or non-repeated clinical data) were examined using patient-level univariate models. To appropriately account for the repeated PET/CT scans per patient and to avoid violating independence assumptions, the multivariable model was re-specified as a mixed-effects logistic regression, with patient ID included as a random intercept. All scan-level predictors were entered as fixed effects. Model fit indices, effect estimates, and 95% confidence intervals were reported for all analyses. A two-sided *p*-value < 0.05 was considered statistically significant.

To address the potential confounding effect of atherosclerosis-related FDG uptake, we performed several prespecified sensitivity analyses. Logistic regression models were repeated after excluding patients aged ≥ 60 years, ever-smokers, and individuals with chronic kidney disease.

## 3. Results

A total of 100 PET/CT images of 77 (F/M: 63/14) patients were involved in the study. Mean age was 43.0 ± 12.9 years and mean disease duration was 120.1 ± 88.8 months. The characteristics of the patients were summarized in Table 1.

**Figure 1:**
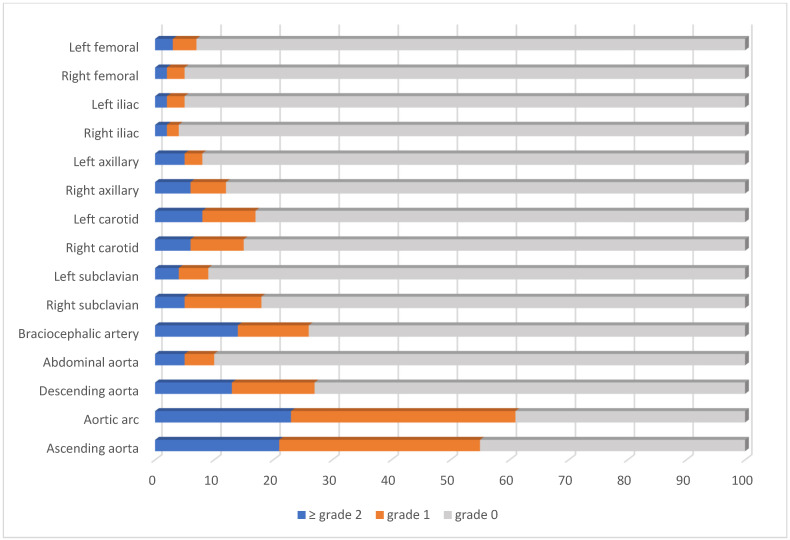
Frequency of FDG uptake of the arterial sites in patients with Takayasu arteritis.

The most frequently involved artery was aortic arc (71%) followed by ascending aorta (55%). The median number of arteries with FDG uptake was 2 (0–15) (Figure 1).

Thirty-nine (51%) patients had type 5 disease according to Hata angiographic classification [12], 34 (44%) patients were classified in cluster 2 and 15 (19%) patients cluster 1 disease according to Goel et al.’s angiographic cluster analysis [13]. The median PETVAS was 3 (0–27) and 31 (31%) PET/CT images were active according to VA. The mean number of atherosclerotic risk factors was 1.86 ± 1.23 in TAK patients.

The iliac and/or femoral arteries were involved in 9 (12%) patients. When the baseline characteristics of patients were compared, the ratio of male patients (44% vs. 6%, *p* = 0.015), the frequency of smoking (77% vs. 40%, *p* = 0.045) and chronic kidney disease (22% vs. 4%, *p* = 0.046) were higher in patients with iliofemoral involvement. Also, the mean age was numerically higher in this group (52.5 ± 17.4 vs. 41.3 ± 12.1, *p* = 0.098). There was no difference between the groups in terms of other atherosclerotic risk factors: diabetes, hypertension, hyperlipidemia, obesity, and family history of atherosclerotic vascular disease (Table 1).

The frequency of patients with active disease was similar between the groups (Active patient ratio iliofemoral involvement + vs. −: 67% vs. 42%, *p* = 0.15). The rate of GC use and immunosuppressives were similar between patients with and without iliofemoral involvement. While CRP levels were higher (median CRP: 38.5 mg/L vs. 10.7 mg/L, *p* = 0.026) in the iliofemoral group, erythrocyte sedimentation rate (ESR) was similar (median ESR: 32 mm/h vs. 35 mm/h, *p* = 0.71). Although NIH/Kerr-defined clinical activity was similar between groups (active patients iliofemoral involvement + vs. −: 67% vs. 42%, *p* = 0.15), PET-based vascular activity was more frequent in iliofemoral positive scans. Active PET according to VA was more frequent in iliofemoral involvement group (67% vs. 27%, *p* = 0.015). PETVAS was also numerically higher in this group, however, the difference did not reach statistical significance (Median PETVAS iliofemoral involvement + vs. −: 7.5 (0–26) vs. 2 (0–17), *p* = 0.05) (Table 2).

Older age (OR (95% CI): 1.07 (1.01–1.14), *p* = 0.031) and male gender (OR (95% CI): 5.80 (1.24–27.05), *p* = 0.025) were associated with iliofemoral artery involvement in univariate analysis. There were also trends toward significance for smoking (OR (95% CI): 4.79 (0.92–24.92), *p* = 0.062), chronic kidney disease (OR (95% CI): 6.00 (0.85–42.26), *p* = 0.072) and CRP levels (OR (95% CI): 1.02 (1.00–1.04), *p* = 0.055) in univariate analysis. In multivariate logistic regression analysis, male gender (OR = 6.68, 95% CI 1.10–40.64, *p* = 0.039) and older age (OR = 1.07, 95% CI 1.002–1.15, *p* = 0.044) were independently associated with iliofemoral involvement (Table 3). The mixed-effects approach produced results consistent with the original multivariable analysis, supporting the robustness of the findings. Male patients had significantly higher odds of exhibiting iliofemoral involvement compared with female patients (B = −1.907, 95% CI −3.714 to −0.101, *p* = 0.038). In addition, older age was modestly but significantly associated with iliofemoral PET positivity (B = 0.072, 95% CI 0.002 to 0.141, *p* = 0.042).

Sensitivity analyses excluding older patients, smokers, and those with chronic kidney disease demonstrated that the direction of associations between clinical variables and iliofemoral FDG uptake remained similar to the main analyses. However, after excluding patients ≥ 60 years, ever-smokers, or those with chronic kidney disease, confidence intervals widened and *p* values became non-significant, consistent with reduced sample size. These results support the robustness of the overall signal while illustrating the known difficulty of differentiating vasculitic and atherosclerotic FDG uptake in iliofemoral arteries (Appendix A Table A1, Table A2 and Table A3).

## 4. Discussion

In this study, we demonstrated that iliofemoral artery involvement on PET/CT is associated with traditional atherosclerotic risk factors in patients with TAK. Older age, male sex, smoking, and the presence of chronic kidney disease were more frequent among patients with iliofemoral artery involvement. Also, patients with iliofemoral involvement had higher CRP levels. These findings suggest that atherosclerotic mechanisms may coexist in TAK, particularly in patients with an unfavorable cardiovascular risk profile.

The overlap between atherosclerosis and TAK has increasingly recognized. Although TAK is a primary large-vessel vasculitis characterized by granulomatous inflammation, the chronic systemic and vascular inflammation promotes oxidative stress, and alters lipid metabolism, leading to endothelial dysfunction and increased atherosclerosis risk [14]. Accelerated atherosclerosis was found more frequent in TAK compared to healthy controls in another study [3]. In our cohort, the association of iliofemoral artery involvement, a hallmark of peripheral atherosclerosis in the general population, with classical cardiovascular risk factors suggests the coexistence of inflammatory and atherosclerotic pathways in TAK.

The contribution of age and gender to the development of atherosclerosis is consistent with previous reports. In TAK, male patients tend to develop more extensive vascular damage, including aneurysmal disease and iliac artery involvement, and more extensive aortic lesions or aneurysms with more complications compared to females [15,16]. Similarly, older age has been associated with a greater atherosclerotic plaque burden in TAK, supporting an age-related contribution to vascular injury [17]. Also, a large multicenter cohort study demonstrated that TAK is associated with considerable long-term vascular morbidity. In a study of 318 patients followed for a median of 6.1 years, relapses occurred in 43% and vascular complications in 38% patients, demonstrating the chronic and progressive nature of vascular injury in TAK [18]. These findings suggest that age-related vascular remodelling and chronic inflammation may jointly promote arterial wall injury and progression of structural damage in TAK. Similarly, older age and male gender were more common among patients with iliofemoral artery involvement in our patients.

Smoking remains a major modifiable risk factor for peripheral vascular disease and atherosclerosis. It contributes to endothelial dysfunction, vascular inflammation, and arterial stiffness [19]. Similarly, in TAK, smoking has been associated with increased mortality and vascular complications [2,20]. The significantly higher smoking rate among our patients with iliofemoral involvement further supports its pathogenic contribution to vascular injury.

Another key finding was the elevated CRP levels among patients with iliofemoral artery involvement. While CRP is often viewed as an inflammation marker, evidence suggests it may also actively participate in atherosclerosis by modulating endothelial cells, smooth muscle proliferation, and lipid uptake [21]. In our study, higher CRP levels in patients with iliofemoral artery involvement may therefore reflect both ongoing vascular inflammation and concomitant atherosclerotic activity.

In our cohort, PGA-based clinical activity did not differ between groups, whereas PET-derived vascular activity (according to VA) was higher in patients with iliofemoral involvement. Such discordance between clinical and metabolic activity has been well described in TAK and likely reflects the fact that PGA captures systemic inflammatory manifestations, while PET/CT detects segmental vascular metabolic activity, which may remain active despite clinically inactive disease.

In many cases, FDG uptake in TAK in vessel walls likely reflects a mix of both active vasculitis and inflamed atherosclerotic plaques. This overlap makes it difficult to tell whether increased PET/CT activity represents inflammation from vasculitis or from atherosclerosis [5]. Thus, iliofemoral FDG uptake should be interpreted cautiously, especially in patients with multiple cardiovascular risk factors. In our study, patients with iliofemoral artery involvement also showed higher PETVAS scores, suggesting that metabolic activity in these regions may not solely indicate active vasculitis but could also reflect concomitant atherosclerotic inflammation. Importantly, PETVAS does not incorporate iliac or femoral arteries. This exclusion is intentional in the original scoring system because lower-limb arteries are highly prone to atherosclerotic FDG uptake, which reduces specificity for vasculitic inflammation. In our study, PETVAS was therefore used to represent global inflammatory activity, whereas iliofemoral uptake—which may reflect both vasculitic and atherosclerotic processes—was evaluated separately. Including these territories within a modified PETVAS could introduce misclassification rather than improve accuracy, particularly in older or high-risk patients.

Sensitivity analyses excluding older patients, smokers, and those with chronic kidney disease showed consistent effect directions but expected loss of statistical significance due to reduction in sample size. These findings suggest that the observed associations are generally robust, while also underscoring the intrinsic challenge of separating atherosclerotic from vasculitic metabolic activity in the iliofemoral region.

A key diagnostic challenge in interpreting iliofemoral FDG uptake is that this region is prone to both atherosclerotic inflammation and vasculitic involvement, and PET/CT alone may not fully distinguish between the two. Although we used pattern-based visual criteria to differentiate circumferential vasculitic uptake from focal or eccentric atherosclerotic activity, some diagnostic uncertainty remains. Therefore, iliofemoral PET activity in this study should be interpreted cautiously, as it may reflect a mixture of atherosclerotic burden and true vasculitic inflammation.

In addition to traditional cardiovascular risk factors, several potential confounders may influence FDG uptake patterns and vascular involvement in TAK. Longer disease duration may contribute to chronic vascular remodelling, cumulative glucocorticoid or immunosuppressive exposure can suppress or modify FDG signal intensity, and varying levels of systemic inflammation (e.g., CRP elevation) may affect metabolic activity independent of atherosclerosis. These factors were not fully controlled for in our retrospective design and may have partially contributed to the observed PET/CT findings. Therefore, the associations identified in this study should be interpreted with caution.

Interestingly, despite higher PET-defined activity, the intensity of immunosuppressive treatment was lower among patients with iliofemoral involvement. This may indicate that iliofemoral uptake was clinically interpreted as atherosclerotic rather than active vasculitic, particularly in older or male patients with cardiovascular risk factors. Supporting this interpretation, six of the nine patients with iliofemoral involvement had PET-active scans, yet treatment was modified in only two of them. Moreover, ongoing glucocorticoid and immunosuppressive therapy may have partially suppressed FDG uptake, suggesting that lower metabolic activity could represent the consequence rather than the cause of reduced treatment intensity. These findings highlight the importance of integrating PET results with clinical and vascular imaging data when evaluating disease activity in TAK.

Interestingly, obesity and dyslipidemia were not associated with iliofemoral involvement in our study. This might be due to the relatively low prevalence of obesity in our cohort and the potential underestimation of metabolic syndrome components in retrospective analyses. Future studies using quantitative lipid and body composition parameters may clarify these relationships.

Beyond metabolic and inflammatory pathways, autonomic dysregulation has been proposed as an additional mechanism contributing to vascular dysfunction in autoimmune diseases. Evidence from systemic lupus erythematosus demonstrates that impaired autonomic reflexes may affect vascular tone and endothelial function, suggesting that similar mechanisms could also be relevant in TAK. Although our study did not evaluate autonomic balance, this represents another potential contributor to vascular heterogeneity in these patients [22].

It is important to emphasize that the associations observed between iliofemoral FDG uptake and atherosclerotic risk factors should not be interpreted as causal. Given the retrospective design, the small number of iliofemoral positive scans, and the inherent limitations of the PET activity assessment, the metabolic signal in these territories may reflect a mixture of vasculitic and atherosclerotic activity. Moreover, dedicated vascular imaging such as duplex ultrasound or calcium scoring was not available to adjudicate atherosclerotic burden in the iliofemoral beds. Therefore, our findings warrant confirmation in larger prospective cohorts with multimodal vascular assessment.

The present study has some limitations. The main limitation of this study is the relatively small sample size, particularly the low number of patients with iliofemoral involvement, which limits the statistical power and may have contributed to the wide confidence intervals in multivariate analyses. Another limitation of the study was the evaluation of PET/CT based on semiquantitative assessment. Third, although visual analysis using the liver-based threshold is widely accepted, this criterion may not reliably distinguish atherosclerotic metabolic activity from vasculitic inflammation, especially in older patients or those with cardiovascular risk factors. Finally, no gold-standard method for confirming atherosclerosis (such as duplex ultrasonography, CT calcium scoring, or CT angiography of iliac/femoral arteries) was available, and therefore we cannot definitively determine whether the observed uptake represents atherosclerosis, vasculitis, or both. These issues require cautious interpretation and highlight the need for prospective studies with standardized vascular imaging. The main strength of this study is that it includes detailed cardiovascular risk information and examines PET findings in lower-limb arteries, an area rarely addressed in TAK.

## 5. Conclusions

In conclusion, our results suggest that iliofemoral FDG uptake may arise from a complex interplay of inflammatory and atherosclerotic processes, particularly in patients with multiple cardiovascular risk factors rather than indicating a direct causal relationship. Involvement in iliofemoral arteries should be interpreted with caution due to the possibility of false positives related to atherosclerotic vascular uptake. Future prospective studies integrating PET/CT, vascular ultrasound, and serum biomarkers are needed to delineate the interplay between inflammation and atherosclerosis in TAK. Finally, our results suggest careful cardiovascular risk assessment and implementation of targeted risk reduction strategies in TAK management.

## Figures and Tables

**Table 1:** The baseline characteristics of Takayasu arteritis patients with and without iliofemoral PET/CT involvement.

	Patients with Iliofemoral Involvement (*n* = 9)	Patients Without Iliofemoral Involvement (*n* = 68)	*p*
Age, years, mean ± SD	52.5 ± 17.4	41.3 ± 12.1	0.098
Gender, male, *n* (%)	4 (44)	4 (6)	0.015 *
Symptom duration, months	64 (48–456)	92 (1–340)	0.98
BMI, kg/m^2^, mean ± SD	23.3 ± 5.6	25.4 ± 4.1	0.39
Angiographic type 5 disease, *n* (%)	4 (44)	35 (51)	0.87
Cluster 1, *n* (%)	2 (22)	13 (19)	0.85
Cluster 2, *n* (%)	3 (33)	31 (46)	0.44
Diabetes, *n* (%)	1 (11)	6 (9)	0.82
Hypertension, *n* (%)	4 (44)	31 (46)	0.94
Hyperlipidemia, *n* (%)	5 (56)	25 (37)	0.27
Smoking, *n* (%)	7 (77)	27 (40)	0.045 *
Obesity, BMI ≥ 30, *n* (%)	0 (0)	8 (12)	0.27
Family history for atherosclerosis, *n* (%)	2 (22)	26 (38)	0.32
Cerebrovascular event, *n* (%)	1 (11)	8 (12)	0.26
Coronary artery disease, *n* (%)	1 (11)	7 (10)	0.96
Chronic kidney disease, *n* (%)	2 (22)	3 (4)	0.046

* Bold values indicate statistical significance (*p* < 0.05).

**Table 2:** Comparison of TAK patients during imagings with and without iliofemoral involvement.

	Images with Iliofemoral Involvement (*n* = 9)	Images Without Iliofemoral Involvement (*n* = 91)	*p*
Treatment			
GC present, *n* (%)	2 (22)	44 (48)	0.11
GC dose *, mg/d	0 (0–7.5)	2.5 (0–80)	0.15
Methotrexate, *n* (%)	1 (11)	11 (12)	0.87
Azathioprine, *n* (%)	2 (22)	28 (31)	0.17
Leflunomide, *n* (%)	3 (33)	15 (17)	0.25
Mycophenolate mofetil, *n* (%)	0 (0)	3 (3)	0.56
TNF inhibitors, *n* (%)	1 (11)	10 (11)	0.95
Tocilizumab, *n* (%)	0 (0)	1 (1)	0.74
TAK disease activity **			
Active disease, *n* (%)	6 (67)	38 (42)	0.15
Inactive disease, *n* (%)	3 (33)	53 (58)	
Laboratory			
ESR, mm/h, mean ± SD	32 (7–120)	35 (4–113)	0.71
CRP, mg/l, median (min-max)	38.5 (3.1–77.4)	10.7 (1.5–126)	**0.026 †**
Imaging			
PETVAS, median (min-max)	7.5 (0–26)	2 (0–17)	0.05
Active PET ***	6 (67)	25 (27)	**0.015 †**

* Prednisolone or its equivalent dose. ** According to PGA. *** According to visual analysis. † Bold values indicate statistical significance (*p* < 0.05). ESR: erythrocyte sedimentation rate, CRP: C reactive protein, PETVAS: PET vascular activity score.

**Table 3:** Logistic regression analysis of the variables associated with iliofemoral involvement.

	Univariate Analysis		Multivariate Analysis	
Variables	O.R. (95% CI)	*p*	O.R. (95% CI)	*p*
Age, years	1.07 (1.01–1.14)	0.031 *	1.07 (1.002–1.15)	0.044 *
Gender, male	5.80 (1.24–27.05)	0.025 *	6.68 (1.10–40.64)	0.039 *
Symptom duration, months	1.00 (0.99–1.01)	0.64	-	
BMI, kg/m^2^	0.89 (0.73–1.07)	0.22	-	
Angiographic type 5 disease	0.88 (0.20–3.84)	0.87	-	
Cluster 1	1.16 (0.21–6.27)	0.86	-	
Cluster 2	0.56 (0.13–2.45)	0.45	-	
Diabetes mellitus	2.95 (0.49–17.52)	0.82	-	
Hypertension	0.95 (0.23–3.86)	0.95	-	
Hyperlipidemia	2.15 (0.52–8.75)	0.29	-	
Smoking	4.79 (0.92–24.92)	0.062	-	
Family history for atherosclerosis	0.43 (0.08–2.33)	0.33	-	
Cerebrovascular event	0.91 (0.10–8.23)	0.93	-	
Coronary artery disease	1.05 (0.11–9.71)	0.96	-	
Chronic kidney disease	6.00 (0.85–42.26)	0.072	-	
Takayasu arteritis disease activity	2.39 (0.55–10.33)	0.25	-	
ESR, mm/h	1.01 (0.99–1.04)	0.35	-	
CRP, mg/L	1.02 (1.00–1.04)	0.055	-	
Presence of GC treatment	0.37 (0.07–1.95)	0.25	-	

* Bold values indicate statistical significance (*p* < 0.05).

## Data Availability

The data presented in this study are available on request from the corresponding author.

## Abbreviations

The following abbreviations are used in this manuscript:

TAK	Takayasu’s arteritis
PETVAS	PET vascular activity score
FDG-PET/CT	18F-fluorodeoxyglucose positron emission tomography/computed tomography
ACR	American College of Rheumatology
PGA	Physician’s Global Assessment
IS	Immunosuppressive
GC	Glucocorticoid
EULAR	European League Against Rheumatism
EANM	The European Association of Nuclear Medicine
SNMMI	The Society of Nuclear Medicine and Molecular Imaging
PIG	PET Interest Group
ASNC	American Society of Nuclear Cardiology
VA	Visual analysis
OR	Odds ratio
CI	Confidence interval

## Appendix A

**Table A1:** Logistic regression analysis excluding patients ≥ 60 years.

Variables	OR (95% CI)	*p*
Age, years	0.97 (0.88–1.07)	0.58
Gender, male	2.33 (0.21–25.66)	0.33
Symptom duration, months	0.99 (0.98–1.01)	0.88
BMI, kg/m^2^	1.07 (0.82–1.40)	0.59
Angiographic type 5 disease	0.68 (0.09–5.25)	0.71
Cluster 1	1.30 (0.12–13.77)	0.82
Diabetes	9.00 (0.62–129.59)	0.11
Hypertension	0.47 (0.04–4.89)	0.53
Hyperlipidemia	0.64 (0.06–6.67)	0.71
Smoking	4.50 (0.43–46.09)	0.20
Family history for atherosclerosis	0.37 (0.03–3.85)	0.41
Cerebrovascular event	2.66 (0.23–29.90)	0.42
Coronary artery disease	Not estimable	0.99
Chronic kidney disease	Not estimable	0.99
Takayasu arteritis disease activity	0.41 (0.04–4.22)	0.45
ESR, mm/h	1.007 (0.97–1.05)	0.70
CRP, mg/L	1.007 (0.97–1.04)	0.70
Presence of GC treatment	1.24 (0.16–9.43)	0.83

**Table A2:** Logistic regression analysis excluding ever-smokers.

Variables	OR (95% CI)	*p*
Age, years	1.10 (0.97–1.24)	0.11
Gender, male	7.33 (0.40–133.28)	0.17
Symptom duration, months	0.98 (0.96–1.01)	0.40
BMI, kg/m^2^	1.36 (0.63–2.91)	0.43
Angiographic type 5 disease	0.75 (0.04–12.98)	0.84
Cluster 1	Not estimable	0.99
Diabetes	Not estimable	0.99
Hypertension	Not estimable	0.99
Hyperlipidemia	2.70 (0.15–47.39)	0.49
Family history for atherosclerosis	Not estimable	0.99
Cerebrovascular event	7.75 (0.40–149.70)	0.17
Coronary artery disease	Not estimable	
Chronic kidney disease	16.5 (0.73–372.80)	0.07
Takayasu arteritis disease activity	Not estimable	
ESR, mm/h	1.06 (0.96–1.18)	0.21
CRP, mg/L	1.02 (0.99–1.05)	0.14
Presence of GC treatment	Not estimable	

**Table A3:** Logistic regression analysis excluding patients with chronic kidney disease.

Variables	OR (95% CI)	*p*
Age, years	1.03 (0.96–1.10)	0.32
Gender, male	2.12 (0.36–12.49)	0.40
Symptom duration, months	1.01 (0.99–1.12)	0.30
BMI, kg/m^2^	0.94 (0.77–1.16)	0.60
Angiographic type 5 disease	0.96 (0.18–5.17)	0.97
Cluster 1	1.89 (0.32–11.03)	0.47
Diabetes	4.64 (0.71–30.32)	0.10
Hypertension	0.53 (0.09–2.96)	0.47
Hyperlipidemia	1.50 (030–7.32)	0.61
Smoking	7.61 (0.86–67.26)	0.06
Family history for atherosclerosis	0.27 (0.03–2.50)	0.25
Cerebrovascular event	1.33 (0.13–12.75)	0.80
Coronary artery disease	Not estimable	
Takayasu arteritis disease activity	1.66 (0.34–8.07)	0.52
ESR, mm/h	1.01 (0.98–1.03)	0.37
CRP, mg/L	1.01 (0.99–1.04)	0.13
Presence of GC treatment	0.52 (0.09–2.91)	0.46
